# HTLV-1 subtype C infection in a socially disadvantaged Indigenous Australian population: epidemiological insights from a retrospective hospital-based cohort study

**DOI:** 10.1186/1742-4690-12-S1-O1

**Published:** 2015-08-28

**Authors:** Lloyd Einsiedel, Richard Woodman, Maria Flynn, Clinton Pepperill, Kim Wilson, Olivier Cassar, Antoine Gessain

**Affiliations:** 1 Flinders University/Northern Territory Rural Clinical School, Alice Springs Hospital, Northern Territory, Australia; 2 National Serological Reference Laboratory, Melbourne, Australia; 3 Institut Pasteur, Unité d'Epidémiologie et Physiopathologie des Virus Oncogènes, Département de Virologie, Paris, France; 4 CNRS, UMR 3569, Paris, France

## 

The Human T-Lymphotropic Virus 1 (HTLV-1) is endemic to central Australia. No attempt has been made to control transmission among Indigenous Australians and no epidemiological studies are available that could direct any intervention. Here we report the epidemiological associations of HTLV-1 infection in a hospital cohort of socially disadvantaged Indigenous adults. Results of HTLV-1 serology were obtained from the Alice Springs Hospital (ASH) pathology database for Indigenous adults admitted 1st January 2000 to 31st December 2013. HTLV-1 proviral loads were determined for 109 asymptomatic adults. Among 1950 Indigenous adults screened, HTLV-1 western blots were positive for 621 (31.8%) and indeterminate for 51 patients (2.6%). Two of 17 (11.8%) children from western desert communities were HTLV-1 infected. Seropositivity rates increased with age and were highest for older men. In a multivariable model, increasing age, male gender, residence in the south or west and having positive syphilis serology were associated with HTLV-1 infection. Three seroconversions were recorded among 351 adults who were tested more than once during the study period. The annual incidence rate of HTLV-1 infection in this sub-group was 0.85 per 100 person-years. HTLV-1 proviral loads were <0.01 per 100 PBMC in 53 (48.6%), and exceeded 1% PBMC in 23 (19.7%) asymptomatic adults. In a hospital-based cohort of Indigenous Australians, we found childhood infections suggestive of mother-to-child transmission, document horizontal transmission and reveal an association with positive syphilis serology in a multivariable model. Cultural practices may have further amplified infection rates among older men. Multiple modes of transmission are therefore likely to contribute to high rates of HTLV-1c infection among the Indigenous population of central Australia. Most asymptomatic adults who were HTLV-1 infected appeared to have good control of HTLV-1 replication.

